# Sample preparation and electrochemical data of Co_3_O_4_ working electrode for seawater splitting

**DOI:** 10.1016/j.dib.2017.07.030

**Published:** 2017-07-14

**Authors:** Malkeshkumar Patel, Wang-Hee Park, Abhijit Ray, Joondong Kim, Jung-Ho Lee

**Affiliations:** a Photoelectric and Energy Device Application Lab (PEDAL), Department of Electrical Engineering, Incheon National University, 119 Academy Rd. Yeonsu, Incheon 406772, Republic of Korea; b Department of Solar Energy, Pandit Deendayal Petroleum University, Raisan, Gandhinagar 382007, Gujarat, India; c Department of Materials and Chemical Engineering, Hanyang University, Ansan, Kyunggido 426-791, Republic of Korea

**Keywords:** Co_3_O_4_ semitransparent film, Porous Co_3_O_4_ working electrode, Kirkendall-diffusion, Sea water splitting

## Abstract

In this data article, we presented the electrochemical data of the working electrode made of Co_3_O_4_ semi-transparent film. Electrochemically stable, porous nature of Kirkendall-diffusion grown Co_3_O_4_ films were applied to generate hydrogen from the seawater splitting (Patel et al., 2017) [1]. The data presented in this article includes the photograph of prepared samples, polarization curves for water oxidation and Tafel plot, linear sweep voltammetry measurements under the pulsed light condition in 0.1 M Na_2_S_2_O_3_ electrolyte, and transient photoresponses with natural sea water. Moreover, seawater splitting using the Co_3_O_4_ working electrode is demonstrated.

## **Specifications Table**

**Table:** 

Subject area	*Physics, Chemistry, Electrical Engineering*
More specific subject area	*Solar Materials, Water Splitting, Hydrogen*
Type of data	*Photograph, Figures, Video*
How data was acquired	*Digital camera* *Potentiostat/Galvanostat (ZIVE, SP1, WonA Tech, Korea)*
Data format	*Video Clip (.avi) and Analyzed*
Experimental factors	*Photograph of Sample: Day light* *Linear sweep voltammetry:* 1. *Polarization curves, scan direction 1.2–2.5 V vs RHE, iR corrected, scan step 5 mV, scan speed 5 mV/s, natural sea water* 2. *Photocathode, scan direction 0.4 V to −0.7 V vs RHE, scan step 5 mV, scan speed 25 mV/s, electrolyte 0.1 M Na*_*2*_*S*_*2*_*O*_*3*_ *Spectral response: Chronoamperometry technique, Applied potential 0.33 V vs RHE* *Light source:365 nm, 2 mW/cm*^*2*^ *460 nm, 3 mW/cm*^*2*^ *520 nm, 6 mW/cm*^*2*^ *620 nm, 15 mW/cm*^*2*^ *Demonstration video:Natural sea water (Yellow Sea near the Incheon National University at 37.3751° N, 126.6328° E coordinate (7 Jun 2016, pH 7.69 (Martini Instruments, pH 56), White light source 100 mW/cm*^*2*^*, current density 20 mA/cm*^*2*^*@ −0.8 V vs RHE*
Experimental features	*Kirkendall diffusion grown porous Co*_*3*_*O*_*4*_*electrode and sea water splitting*
Data source location	*Incheon National University, Incheon 22012, Korea*
Data accessibility	*The data are with this article*

## **Value of the data**

• Photographs of the Co_3_O_4_ samples for the large working area of 60 mm^2^.
• Polarization curves for water oxidation can be useful to design the water splitting research.
• Linear sweep voltammetry of Co_3_O_4_ working electrode in 0.1 M Na_2_S_2_O_3_ electrolyte examined for photocathode properties
• Spectral responses of Co_3_O_4_/FTO photocathode with natural seawater at over potential of 0.33 V vs RHE are promising for photo-catalyst water splitting application.

## 1 Data

**Fig. 1:**
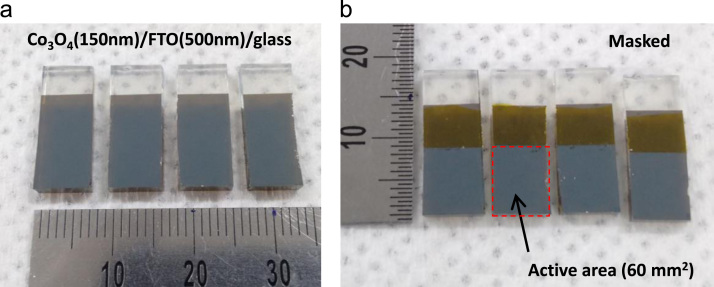
Photographs of the developed porous Co_3_O_4_ films on the FTO/glass substrate. (a) as prepared 150 nm thick Co_3_O_4_ layer on the 500 nm thick FTO-coated glass. (b) Co_3_O_4_ films were masked using the Kapton tape so that 60 + 0.5 mm^2^ of the planar area remained for contacting the electrolyte (natural seawater).

**Fig. 2:**
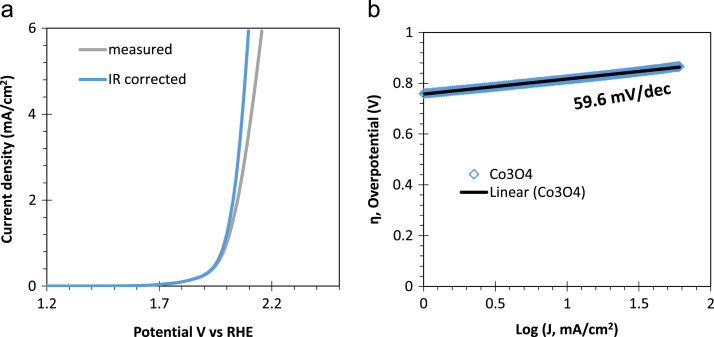
Electrochemical characterization of the Co_3_O_4_ electrode for the anodic potential. (**a**) Current-voltage characteristics and (**b**) Tafel plot derived from (a).

**Fig. 3:**
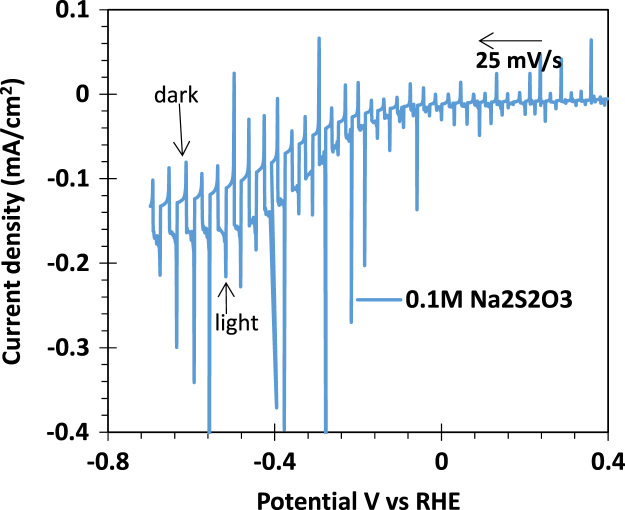
LSV measurement of porous Co_3_O_4_ electrodes under a pulsed light condition with 0.1 M Na_2_S_2_O_3_ electrolyte.

**Fig. 4:**
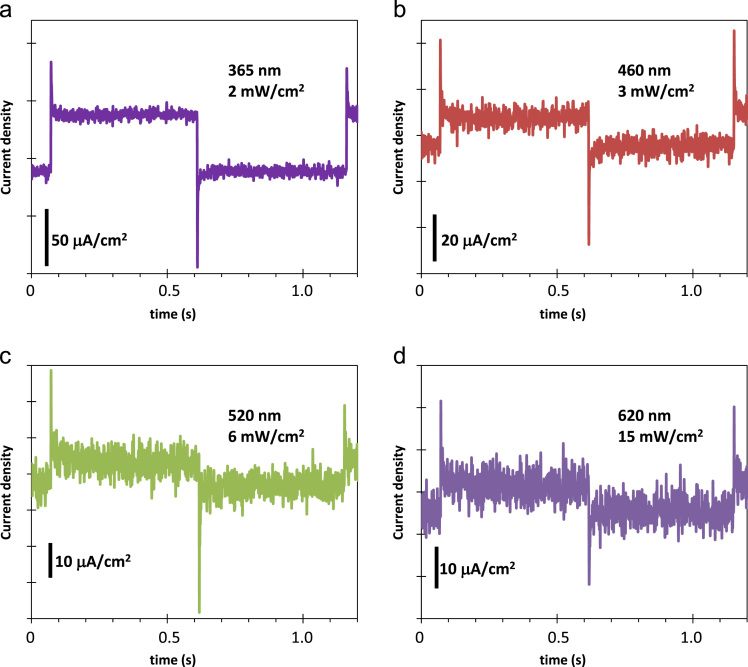
Spectral response of Co_3_O_4_/FTO photocathode with natural seawater showed fast transient at applied over potential 0.33 V vs RHE.

Fig. 1 shows the photographs of the developed porous Co_3_O_4_ films on the FTO/glass substrate. Co_3_O_4_ films were masked using Kapton tape to define the working area (60 mm^2^) for electrochemical experimentation are shown in Fig. 1b. Polarization curve for water oxidation cycle (as shown in Fig. 2) are presented for the natural sea water. Tafel analysis is presented in Fig. 2b. Linear sweep voltammetry (LSV) measurement of the porous Co_3_O_4_ electrode was measured under a pulsed light (100 mW/cm^2^) condition with 0.1 M Na_2_S_2_O_3_ electrolyte in cathodic direction, as shown in Fig. 3. Fig. 4 shows the spectral photoresponse of Co_3_O_4_/FTO photocathode with natural sea water at over potential of 0.3 V vs RHE. Photoinduced seawater splitting using the porous Co3O4 working electrode is demonstrated in the moving clip. In this video current density is 20 mA/cm^2^ @ −0.8 V vs RHE.

## 2 Experimental design, materials and methods

Preparing Co_3_O_4_ electrode: Co3O4 working electrodes were prepared using the Kirkendall diffusion method [1]. Initially the sputtered pure Co film was grown for the porous and semitransparent Co_3_O_4_ film by the heat treatment (550 °C for 10 minutes in air condition). Photographs of Co3O4 samples are shown in Fig. 1.

Electrolytes:

1. Natural seawater: *Yellow Sea near the Incheon National University at 37.3751° N, 126.6328° E coordinate (7 Jun 2016, pH 7.69 (Martini Instruments, pH 56), White light source 100 mW/cm*^*2*^*, current density 20 mA/cm*^*2*^ *@ −0.8 V vs RHE*
2. 0.1 M Na_2_S_2_O_3_: Aqueous electrolyte (100 ml) was prepared from the laboratory grade Na_2_S_2_O_3_

Electrochemical Measurements: All measurements were done using the three electrode electrochemical cells (Reference electrode: Ag/AgCl (KCl, 3 M), Counter electrode: platinum gauze, and working electrode: Co_3_O_4_/FTO/glass) attached to the Potentiostat/Galvanostat (PG-stat) (WonA Tech, ZIVE SP1). Linear sweep voltammetry was applied to measure the anodic polarization and photocathode properties. Chronoamperometry technique was applied to measure the transient photoresponses at an applied potential of 0.33 V vs RHE in the natural seawater. The white light (5800 K, Bridgelux, ES Star Array, BXRA-56C0700-A) was applied for photoinduced electrochemical measurement. This was calibrated by a power meter (KUSAMMECO, KM-SPM-11). The illuminating light source was calibrated for one-sun light intensity (100 mW/cm^2^) and was applied in the pulse mode or the continuous mode. For transient photoresponses, a monochromatic light source of wavelength 365 nm (2 mW/cm^2^), 460 nm (3 mW/cm^2^), 520 nm (6 mW/cm^2^), and 620 nm (15 mW/cm^2^) were applied to working electrode from the front direction.
